# S-Layer Protein Self-Assembly

**DOI:** 10.3390/ijms14022484

**Published:** 2013-01-25

**Authors:** Dietmar Pum, Jose Luis Toca-Herrera, Uwe B. Sleytr

**Affiliations:** Department of Nanobiotechnology, Institute for Biophysics, University of Natural Resources and Life Science, Vienna, Muthgasse 11, Vienna 1190, Austria; E-Mails: jose.toca-herrera@boku.ac.at (J.L.T.-H); uwe.sleytr@boku.ac.at (U.B.S.)

**Keywords:** S-layer, self-assembly, fusion protein, surface functionalization, nanobiotechnology

## Abstract

Crystalline S(urface)-layers are the most commonly observed cell surface structures in prokaryotic organisms (bacteria and archaea). S-layers are highly porous protein meshworks with unit cell sizes in the range of 3 to 30 nm, and thicknesses of ~10 nm. One of the key features of S-layer proteins is their intrinsic capability to form self-assembled mono- or double layers in solution, and at interfaces. Basic research on S-layer proteins laid foundation to make use of the unique self-assembly properties of native and, in particular, genetically functionalized S-layer protein lattices, in a broad range of applications in the life and non-life sciences. This contribution briefly summarizes the knowledge about structure, genetics, chemistry, morphogenesis, and function of S-layer proteins and pays particular attention to the self-assembly in solution, and at differently functionalized solid supports.

## 1. Introduction

Many strains of Eubacteria and Archaea contain regular protein arrays as the outermost component of their cell envelopes [1–6]. These crystalline arrays are commonly referred to as surface layers, or S-layers, and have to be considered as one of the most abundant prokaryotic cellular proteins [1,7,8]. S-layers are generally composed of a single molecular species, protein or glycoprotein in nature (*M*_r_ 40 to 200 kDa), which are endowed with the ability to self-assemble by an entropy-driven process into two-dimensional arrays, both in the presence and absence of surfaces suitable for adhesion [3,7–12].

The self-assembly property is one of the key features of S-layer proteins and, in combination with the precise repetitive exposition of functional groups, laid foundation for the development of a unique biomolecular construction kit [7,10,12]. In comparison to the well-known self-assembled monolayers, e.g., made of alkane thiols on gold, the size of S-layer unit cells is in the range of 10 nm and thus provides a repetition of functional groups suitable for dense binding of many biological molecules. Further on, the design and expression of specific genetically engineered S-layer fusion proteins opened the doors to a completely new approach in the development of novel affinity matrices, the controlled immobilization of molecules and nanoparticles, or the synthesis of nanomaterials [8,13,14]. This review summarizes the key features of S-layer proteins with a particular focus on their self-assembly into 2D crystals in solution and at interfaces.

## 2. Occurrence and Location of S-Layers

**Figure 1:**
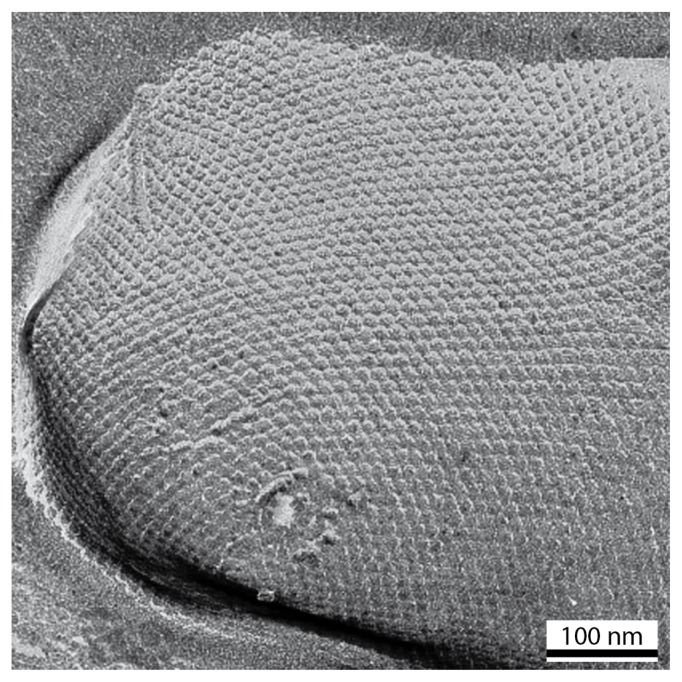
Transmission electron micrograph of a freeze-etched and metal shadowed preparation of an archaeal cell (*Methanocorpusculum sinense*) exhibiting an S-layer with hexagonal lattice symmetry [21].

S-layers are found in members of nearly every taxonomic group in Gram-positive and Gram-negative bacteria and archaea, and some green algae (Figure 1) [3,8,15–18]. Crystalline layers similar to S-layers have also been detected in bacterial sheaths and spore coats [19,20]. On the basis of structural and biochemical studies, S-layer carrying cell envelope profiles can be classified into three main categories, namely (i) archaeal cell envelopes, that are composed of a cytoplasmic membrane and an associated S-layer, (ii) Gram-positive cell envelope profiles, where the S-layer is either attached to a thick and rigid peptidoglycan-containing layer or another polymer (e.g., pseudomurein external to the cytoplasmic membrane), and (iii) Gram-negative cell envelopes, where the S-layer is attached to the outer membrane which is bound to a thin peptidoglycan sacculus.

Since S-layer proteins are produced in larger amounts than any other class of proteins in the cell, they represent remarkably simple model systems for studying the processes involved in the synthesis, glycosylation, and secretion of extracellular proteins [11,22]. It must be stressed here that S-layer proteins are one of the most abundant biopolymers on Earth (it is assumed that 2/3 of the total biomass may be allocated to prokaryotic organisms and that *ca.* 10% may be assigned to S-layer proteins) [15]. In addition, it may be assumed that *ca.* 500.000 S-layer monomers are required for covering a rod-shaped bacterial cell completely. Many S-layer carrying bacteria can grow with generation times of less than 20 min, necessitating the synthesis of more than 400 copies of a single polypeptide chain per second.

Studies on the structure-function relationship of different S-layers from Bacillaceae revealed the existence of specific binding domains on the *N*-terminal part of S-layer proteins for hetero polysaccharides (secondary cell wall polymers), covalently linked to the peptidoglycan matrix of the cell wall [15]. In the following, S-layer-homology (SLH) motifs, consisting of 50 to 60 amino acids each and being responsible for the anchoring, have been identified on the *N*-terminal or *C*-terminal part of many S-layer proteins [22–30].

Although a considerable amount of knowledge has already been accumulated regarding the structure, chemistry, and morphogenesis of S-layer proteins, relatively little is known about their specific function for the bacterial or archaeal cell [3,7,22]. S-layers are directly involved in the interactions between the cell and its environment. S-layers are frequently lost upon cultivation under laboratory conditions indicating that they provide the organism with an advantage of selection in their natural, competitive habitats. In addition, in some archaea, the S-layer is the only wall component and, as such, acts as a flexible corset, shaping the protoplast [11,20,21].

## 3. Ultrastructure of S-Layers

S-layers cover the cell surface completely. In rod-shaped cells the lattices are fairly uniform and characteristically aligned over the cylindrical part, but exhibit a random orientation and numerous lattice faults at the cell poles and septation sites since crystalline layers cannot cover spherical surfaces by simple bending (Figure 1) [1,6,20,21]. The analysis of the number and distribution of lattice faults led to the conclusion that the constituent units are incorporated at specific sites, and that the S-layer continuously recrystallizes during cell growth, maintaining an equilibrium of lowest-energy [2,3,6,7,11,21,31,32].

S-layers exhibit oblique (p1, p2), square (p4), or hexagonal (p3, p6) lattice symmetry with unit cell dimensions in the range of 3 to 30 nm. Depending on the lattice type, one morphological unit consists of one, two, three, four, or six identical proteins (subunits). In these five, two-dimensional plane groups, only *n*-fold rotation axis (*n* = 1, 2, 3, 4, 6) and the translation are allowed as symmetry operators since the handedness of the protein molecules (chirality) does not allow the appearance of mirror- and glide planes, or inversion centres [33]. Bacterial S-layer lattices are generally five to 20 nm thick, whereas S-layers of archaea reveal a thickness of up to 70 nm [34–36]. S-layers generally represent highly porous protein meshworks (30%–70% porosity) with pores of uniform size and morphology in the two to eight nm range [37–39].

High-resolution electron and scanning force microscopic studies revealed a smooth topography for the outer face of most S-layers and a more corrugated one for the inner face [34–36]. Concerning the physicochemical properties of S-layers in *Bacillacaea*, it was demonstrated that the outer face is usually charge neutral, while the inner one is often net negatively or positively charged [39–42]. The surface charge depends on the equity or excess of exposed carboxylic acid or amine groups. Functional groups on the S-layer lattice are aligned in well-defined positions and orientations, which are key for binding molecules and nanoparticles into ordered arrays at these protein lattices [7,11,12,41,43,44].

## 4. Genetic Engineering of S-Layer Proteins

Structure-function relationship of distinct amino acid segments of different S-layer proteins were investigated in order to gain knowledge about those positions where foreign peptide sequences can be fused without disturbing the self-assembly properties. For example, in the case of the S-layer protein SbsB from *Geobacillus stearothermophilus* PV72/p2, minimum-sized core-streptavidin (118 amino acids) could be fused to the *N*- or *C*-terminal end [45]. The fusion proteins and core-streptavidin were produced independently in *Escherichia coli*, isolated, purified, and refolded to heterotetramers consisting of one chain of *N*- or *C*-terminal SbsB-streptavidin fusion protein and three chains streptavidin. The biotin binding capacity of the hetero tetramers was ~80% in comparison to homo tetramers. These findings indicated that at least three of the four streptavidin residues were accessible and active for binding biotinylated molecules. Such chimaeric S-layer fusion proteins can be used as versatile templates for arranging any biotinylated compounds on the outermost surface of the protein lattice.

In a similar approach, the structure-function relationship of the S-layer protein SbpA of *Lysinibacillus sphaericus* CCM2177 was investigated [46–48]. As described above, the final goal was to construct fusion proteins with the ability to reassemble into two-dimensional arrays while presenting the introduced functional sequence or domain on the outermost surface of the protein lattice for binding molecules (see Table 1). It must be noted that the *C*-terminally truncated form rSbpA_31–1068_, which is 1038 amino acids long, is most often used as a basic molecular building block for making various S-layer fusion proteins.

**Figure 2:**
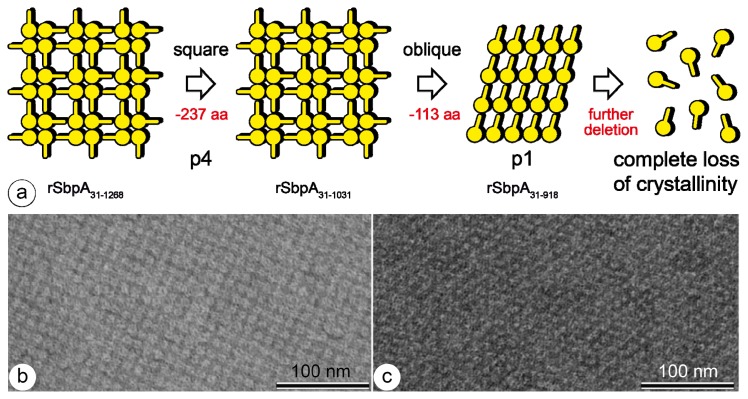
(**a**) Schematic drawing of the conversion of the S-layer lattice symmetry of SbpA, from square to oblique, and complete loss of crystallinity; (**b**) TEM image of the rSbpA_31–1268_ lattice showing square, and (**c**) of the rSbpA_31-918_ lattice exhibiting oblique lattice symmetry.

While screening various truncated forms of rSbpA for their ability for reassembly, it was found that further deletion of 113 *C*-terminal amino acids from rSbpA_31–1031_, leading to rSpbA_31–918_, had a strong and unexpected impact on lattice formation [24]. Contrary to the original S-layer lattice formed by the mature and the truncated form rSbpA_31–1031_ exhibiting square symmetry with a lattice constant of 13.1 nm, a lattice with oblique lattice symmetry and base vectors of *a* = 10.4 nm and *b* = 7.9 nm, and a base angle of 81° was formed (Figure 2). It is interesting to note that the ultrastructure of this newly formed S-layer lattice was identical to that of SbsB, the S-layer protein of *G. stearothermophilus* PV72/p2 [45]. The mature SbsB comprises amino acids 32 to 920 and was only one amino acid longer than rSbpA_31–918_. Both S-layer proteins carry three SLH-motifs on the *N*-terminal part which showed high identity. However, no sequence identities were found for the middle and *C*-terminal parts. Further *C*-terminal truncation of rSbpA_31–918_ led to a complete loss of the self-assembly properties of the S-layer protein.

## 5. Reassembly of S-Layer Proteins

### 5.1. Isolation Procedures

Most techniques for isolation and purification of S-layer proteins involve a mechanical disintegration of cells, and subsequent differential centrifugation to separate the cell wall fragments [6,31,49,50]. With gram-positive organisms, the crude cell wall preparations are frequently treated with detergents, such as Triton X-100, to dissolve the plasma membrane contaminants. S-layer fragments have been obtained either by digestion of the peptidoglycan layer with lysozyme, or by treatment with low concentrations of chaotropic agents (e.g., 0.5 M urea, 1–2 M guanidine hydrochloride (GHCl)), both of which loosen the bonds to the supporting polymer layer without dissociating the S-layer lattice. A complete disintegration of S-layer fragments has been achieved by high concentrations of urea or GHCl, by action of chelating agents, or by changing the pH. With gram-negative eubacteria, S-layers have been removed from the outer membrane of isolated cell envelope fragments through several procedures, including treatment with low concentrations of urea or GHCl, metal chelating agents (e.g., EDTA, EGTA), SDS, cation substitution, and detergents or combinations of them. In archaea, special isolation and purification procedures for the S-layers, including treatments with Triton X-100 and SDS, changes in pH and ionic strength, or extraction with organic solvents, have been applied [50]. In addition, it has also been shown that recombinant S-layer proteins may be secreted into the culture medium and were able to form self-assembly products in suspension but did not recrystallize on the surface of the cells [51].

**Figure 3:**
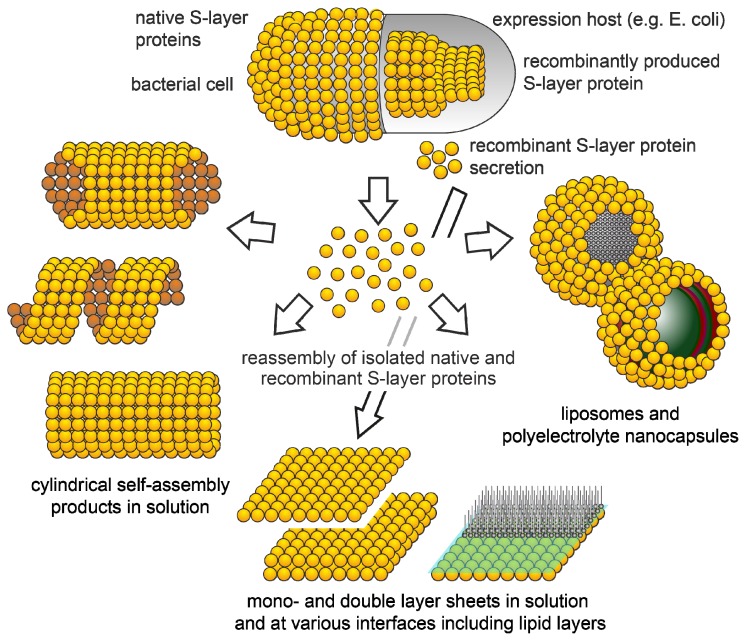
Schematic drawing showing the reassembly of S-layer proteins in solution, on solid supports, at the air-water interface, at lipid films, and at liposomes and nanocapsules.

Reassembly of the isolated S-layer proteins into two-dimensional arrays occurs upon dialysis of the disrupting agents (Figure 3). The formation of the self-assembled arrays is only determined by the amino acid sequence of the polypeptide chains, and consequently the tertiary structure of the S-layer protein species. Since S-layer proteins have a high proportion of non-polar amino acids, most likely, hydrophobic interactions are involved in the assembly process. Some S-layers are stabilized by divalent cations, such as Ca^2+^, interacting with acidic amino acids [52–55]. Studies on the distribution of functional groups, on the surface, have shown that free carboxylic acid groups and amino groups are arranged in close proximity and thus contribute to the cohesion of the proteins by electrostatic interactions [41,56].

Summarizing these detachment and disintegration experiments, it may be concluded that, in eubacteria, S-layer proteins are non-covalently linked to each other and to the supporting cell wall component, differing combinations of weak bonds (hydrophobic bonds, ionic bonds involving divalent cations or direct interaction of polar groups, and hydrogen bonds) are responsible for the structural integrity of the S-layer lattice, and for their adhesion to the underlying cell envelope component. Further on, the bonds holding the S-layer proteins together must be stronger than those binding them to the underlying cell envelope layer [32,57,58].

### 5.2. Reassembly in Solution

Depending on the morphology and bonding properties of the subunits, flat sheets, open-ended cylinders, or closed vesicles can be the final products of the assembly process [7,11]. In some cases it was possible to obtain different assembly products by simply changing the assembly conditions such as pH, temperature, ionic strength, and the presence or absence of divalent cations [59]. In general, the reassembly starts with a rapid initial phase, in which oligomeric precursors are formed, followed by a slow rearrangement step leading to extended lattices [60]. In addition, depending on the S-layer proteins used and the environmental conditions (e.g., ionic content and strength), the formation of double layers may be induced (or avoided) [11,59]. In double layers, the constituent monolayers are commonly not in register, and, to our knowledge, always oriented face-to-face to each other.

### 5.3. Reassembly on Mica and on Silicon Substrates

**Figure 4:**
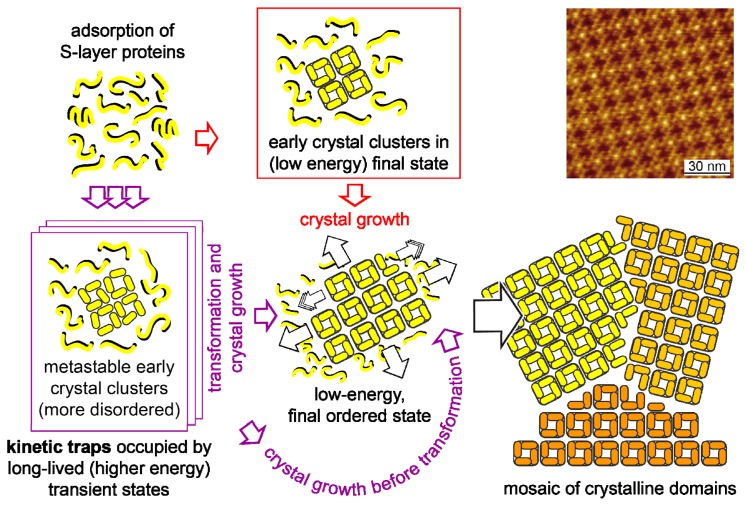
Schematic drawing of the reassembly pathways of the S-layer protein SbpA from *L. sphaericus* on solid surfaces (drawn after description in reference [64]). Inset: AFM image of the S-layer of *L. sphaericus*.

Crystal growth at interfaces is initiated simultaneously at many randomly distributed nucleation points, and proceeds in plane until the crystalline domains meet, thus leading to a closed, coherent mosaic, of individual, several micro meters large, S-layer domains [61–64]. In a recently carried out study, it was shown that the reassembly of SbpA S-layer proteins on mica does not follow the classical pathways of crystal growth. The reassembly is determined by a kinetic trap, associated with conformational differences between a long-lived transient state and the final stable state. Over time, the trapped state also transforms into the final, low-energy, stable state (Figure 4).

The formation of coherent crystalline arrays strongly depends on the S-layer protein species, the environmental conditions of the bulk phase (e.g., temperature, pH, ion composition and ionic strength), the concentration of monomers, and, in particular, on the surface properties of the substrate (hydrophobicity, and surface charge). The growth of S-layers composed of large coherent domains is favoured at low monomer concentrations due to the corresponding low number of nucleation sites. As required by technological demands, a great variety of supports, differing in their physico-chemical properties are currently investigated. Silicon and metal surfaces are exploited for applications in nano electronics, glasses in nano optics, and polymeric surfaces, such as SU-8 resist, in microfluidics. In addition, thin film and carbon coated electron microscope grids (EM-grids), and mica are often used for basic research. In most cases the surface has to be rendered hydrophilic or hydrophobic by plasma treatment before use. For example, the S-layer protein SbpA from *L. sphaericus* CCM2177, which is, currently, one of the most used S-layer proteins for functionalizing solid supports, forms monolayers on hydrophobic silicon supports, and double layers on hydrophilic supports. In addition, in comparison to hydrophilic surfaces, the layer formation is much faster on hydrophobic supports starting from many different nucleation sites and thus leading to a mosaic of small crystalline domains (often referred to as crazy paving) (see also next chapter) [32].

A more sophisticated approach makes use of secondary cell wall polymers (SCWPs) for modifying the surface properties of the support (biomimetic support). According to the orientation on the bacterial cell, on SCWP coated supports, the corresponding S-layer proteins reassemble with their inner faces (*N*-terminus) on the support and thus expose their outer faces towards the environment. This is especially important when functional *C*-terminal S-layer fusion proteins are used for reassembly on solid supports [45,48,65–67].

### 5.4. Reassembly on Self-Assembled Monolayers

In addition to the work described above, a detailed study with a particular focus on the reassembly at different hydrophobic substrates was carried out by varying the protein-substrate interactions and investigating the process in real time through Atomic Force Microsopy (AFM) [68]. Silicon substrates were modified with aminopropyltriethoxysilane (APTS) and octadecyltrichlorosilane (OTS). AFM and quartz-crystal microbalance with dissipation (QCM-D) measurements showed that the substrate hydrophobicity had no effect on the S-layer lattice parameters, the protein layer thickness, or the final protein mass adsorbed per unit area (1700 ng cm^−2^). It was found that hydrophobic surfaces (APTS and OTS) led to faster protein adsorption than silicon dioxide rendered hydrophilic by plasma treatment. AFM measurements showed that the crystalline domains were much smaller on silanized substrates compared to hydrophilic silicon dioxide ones. The combination of AFM and QCM-D demonstrated that S-layer protein crystal formation took place in three steps: nucleation, growth (self-assembly), and domain reorganization. Experiments at different S-layer protein concentrations indicated that protein adsorption was diffusion controlled until a threshold concentration of 0.05 mg mL^−1^ for silanized substrates and 0.07 mg mL^−1^ for silicon dioxide was reached.

In another set of experiments [69] the reassembly of the S-layer protein SbpA, again using AFM and QCM-D, was studied on different self-assembled monolayers carrying methyl (CH_3_), hydroxyl (OH), carboxylic acid (COOH), and mannose (C_6_H_12_O_6_) as terminating functional groups. It was found that the protein adsorption rate and the size of the crystalline domains were influenced by the introduced surface chemistry and protein concentration. It was confirmed that electrostatic interactions (COOH functional groups) induce a faster adsorption than hydrophobic (CH_3_ groups) or hydrophilic (OH groups) interactions. In this study, the shear modulus and the viscosity of the reassembled S-layer on CH_3_C_11_S, CH_3_C_6_S, and COOHC_11_S substrates were also quantified [69]. The shear modulus and the viscosity did not vary as a function of surface chemistry and pH, leading to the conclusion that protein-protein interactions are responsible for the mechanical stability of the formed S-layer.

Finally, the last set of experiments was carried out with self-assembled monolayers (SAMs) demonstrating that the S-layer protein SbpA was sensitive to nanoprotrusions caused by the interplay between hydrophobic and hydrophilic interactions. This could be achieved by using disulfide SAMs with different end groups (OH *versus* CH_3_) and adjusting the lengths of the individual methylene chains [63]. The formation of monolayers was observed when the hydrophobic end groups (CH_3_) surmounted the hydrophilic (OH) ones. In addition, the unit cell size was increased by *ca.* 2 nm. On the contrary, double S-layers were formed when hydrophilic (OH) groups superseded the hydrophobic (CH_3_) end groups. The lattice parameters of the native S-layer were maintained. The threshold for the transition between native and non-native S-layer parameters was four methylene groups.

### 5.5. Reassembly on Polyelectrolyte Layers

In general, S-layer proteins have a certain affinity to biopolymers, in particular to secondary cell wall polymers, which are controlled though carbohydrate-protein interactions. Following this idea, reassembly experiments with S-layer proteins have been performed with the goal to engineer biomimetic surfaces. The first work concerning the reassembly of the S-layer protein SbpA on synthetic polymers [70] already demonstrated that cationic and anionic polyelectrolyte layers are suitable substrates. SbpA-green fluorescent fusion protein (rSbpA-EGFP) reassembled on flat substrates and polymeric capsules and was studied through atomic force microscopy, neutron reflectometry, zeta potential measurements, and confocal microscopy. Different polyelectrolytes were used to functionalize flat surfaces and to create hollow capsules: poly-ethylenimine (PEI), poly-sodium 4-styrenesulfonate (PSS), poly-allylamine hydrochloride (PAH), poly-acrylicacid (PAA), and poly-diallyldimethyl ammonium chloride (PDADMAC). The recrystallization behavior of these S-layer proteins was also investigated under different ionic conditions. It was found that S-layer protein reassembly took place in the presence of CaCl_2_ (and MgCl_2_) on negatively charged polyelectrolytes (PSS and PAA), and on strongly positively charged polyelectrolytes (PDADMAC). However, despite the charged nature of a PAH surface, the recrystallization process led to a disorderly adsorption, with no clear crystalline patterns, probably due to the hydrophobic character of the polyelectrolyte. The lattice parameters of the native S-layer were maintained and a protein thickness of about 14 nm was determined through neutron reflectometry studies. Confocal microscopy demonstrated that the attachment of rSbpA-EGFP onto hollow polyelectrolyte capsules did not shift the fluorescence emission of the chromophore. Thus, it was concluded that the reassembly process did not interfere with the functional part of the S-layer fusion proteins.

A second work concerning the reassembly of the S-layer protein SbpA on cationic or anionic polyelectrolytes allowed to investigate the affinity of the inner and outer surface of the S-layer towards the electrolytes [71]. In this study supramolecular, sandwich-like structures, composed of polyelectrolyte/S-layer/polyelectrolyte, were investigated. It was found that only cationic PAH showed strong affinity to the exposed S-layer surface. Furthermore, it was observed that a compression of about 20 nN was enough to unfold reassembled S-layer proteins on anionic PSS.

Considering the two described studies, S-layer protein SbpA reassembled on anionic terminated PSS allowed the determination of adsorbed protein, the mechanical properties as a function of temperature and the amount of bound water in the whole supramolecular structure [72]. This was achieved by combining AFM, QCM-D, and neutron reflectometry. The results indicated that the protein adsorption on PSS was about 1600 ng cm^−2^ that corresponds to a thickness of *ca.* 14 nm. In the course of the reassembly process, the native S-layer lattice was formed. It was found, that at 55 °C the crystalline pattern of the S-layer was lost, although the protein remained attached to the polymer substrate. The S-layer structure could not be recovered by decreasing the temperature again, and thus it was concluded that the process was irreversible. The mechanical studies showed that typical unfolding forces for protein motifs were in a range of 200 to 700 pN. Furthermore, the combination of the results from QCM-D and neutron reflectometry experiments permitted the calculation of the S-layer density (*ca.* 1.16 g cm^−3^), and an estimation of the amount of bound water. It may be concluded that SbpA forms a loose layer on anionic PSS incorporating a water volume fraction of about 68%.

### 5.6. Reassembly at Lipid Interfaces

The reassembly of S-layer proteins at the air/water interface and on lipid films, and the handling of such layers by standard Langmuir Blodgett (LB) techniques, opened a broad spectrum of applications in basic and applied membrane research [73,74]. It has to be stressed that in archaeal cell envelope structures, which are exclusively composed of an S-layer and a closely associated plasma membrane, this concept of a protein supported lipid layer has optimized over billions of years of evolution. Many of these archaea live under extreme environmental conditions, such as pH < 0.5, and under hydrostatic pressure at temperatures up to 120 °C. S-layer supported LB films show a much higher mechanical robustness and life-time compared to unsupported lipid membranes (e.g., black lipid membranes) [73]. The stabilizing effect of S-layers is primarily explained by a reduction or inhibition of disruptive horizontal vibrations of the lipid molecules. Since fluidity and local order of the lipid molecules are modulated by the repetitive pattern of functional domains of the S-layer, the term “semifluid membrane model” has been coined for this layered composite architecture [52]. Fluorescence recovery after photo bleaching (FRAP) measurements demonstrated that the mobility of the unbound molecules was higher than in other model systems, such as hybrid bilayers or dextran-supported bilayers, due to the gained space made available by the S-layer bound molecules [75]. Neutron and X-ray reflectivity studies clearly indicated that the S-layer protein had not penetrated or ruptured the lipid monolayer [76–79].

**Figure 5:**
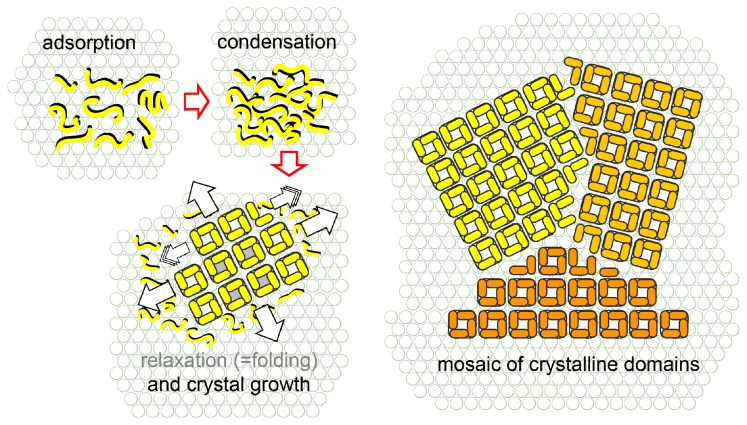
Schematic drawing of the reassembly pathway of the S-layer protein SbpA from *L. sphaericus* on supported lipid films (drawn after description in ref. [80]).

Atomic force microscopy was used to study the reassembly of S-layer proteins into monolayers on supported lipid bilayers (Figure 5) [80]. The reassembly followed a multistage, non-classical pathway in which monomers, with extended conformation, first formed a mobile adsorbed phase from which they condensed into amorphous clusters. In a subsequent phase transition, the S-layer proteins folded into clusters of compact tetramers. In the following, crystal growth proceeded by formation of new tetramers exclusively at cluster edges.

Functional molecules such as ion channels or proton pumps may be incorporated into S-layer stabilized lipid layers, applying well established procedures, and the whole system may be characterized by well established biophysical methods, such as electrophysiology [81–83]. In comparison to plain lipid bilayers, S-layer supported lipid membranes have a decreased tendency to rupture and allow to perform single pore recordings [84].

### 5.7. Reassembly at Liposomes and Nanocapsules

Further on, the reassembly of S-layer proteins on liposomes has great technological importance. Because of their physicochemical properties, liposomes are widely used as model systems for biological membranes, and as delivery systems for biologically active molecules. The presence of S-layer lattices significantly enhanced the stability of the liposomes against mechanical stresses such as shear forces or ultrasonication, and against thermal challenges [70,72,85–88]. Furthermore, S-layer liposomes resemble the supramolecular envelope principle of a great variety of human and animal viruses and, thus, will allow the investigation of artificial viruses, as discussed for gene therapy.

## 6. Summary

S-layer proteins are one of the most abundant biopolymers on Earth, and also the simplest biological membranes developed in the course of evolution. Basic and applied research have led to an accumulated, deep understanding of the structure, genetics, chemistry, morphogenesis, and function of S-layer proteins and their reassembly into lattices with perfect long range order, either in solution or at interfaces. Recently X-ray diffraction studies have been able to solve the atomistic structure of selected S-layer proteins [54,89,90] and 3D structure predictions based on the mean force approach [91–93] allowed to simulate the self-assembly process by Monte-Carlo simulations [94]. These findings will allow us to learn more about the function of S-layers as the outermost bacterial cell envelope component. In summary, it may be anticipated that the broad range of knowledge regarding the basic properties of S-layer proteins provide a profound basis for further basic research, as well as for applied research in the life and non-life sciences.

## Figures and Tables

**Table 1:** Overview of functional domains fused to S-layer proteins and their application [47,48].

Functionality	Application
Core streptavidin	Binding biotinylated ligands (DNA, protein), Biochip development
Major birch pollen allergen (Bet v1)	Vaccines, treatment of type 1 allergy
*Strep*-tag II, Affinity tag for streptavidin	Biochip development
ZZ, IgG-binding domain of Protein A	Extracorporeal blood purification
Enhanced green fluorescent protein (EGFP)	Liposomes, Drug and delivery systems
cAb, Heavy chain camel antibody	Diagnostic systems and sensing layer for labelfree detection systems
Hyperthermophilic enzyme laminarinase (LamA)	Immobilized biocatalysts
Cysteine mutants	Building of nanoparticle arrays
Mimotope of an Epstein-Barr virus (EBV) epitope (F1)	Vaccine development
*M. tuberculosis* antigen (mpt64)	Vaccine development
IgG-Binding domain of Protein G	Downstream processing
Glucose-1-phosphate thymidylyltransferase (RmlA)	Immobilized biocatalysts
Enhanced cyan (ECFP), green (EGFP), yellow (YFP), monomeric red (RFP1) fluorescent protein	pH biosensors *in vivo* or *in vitro*, fluor. markers for drug delivery systems
Metal, silica and titania precipitating peptides	Material sciences
